# Complete Genome Sequence of Vibrio harveyi Strain ATCC 33866

**DOI:** 10.1128/mra.00073-22

**Published:** 2022-05-31

**Authors:** Yue Yao, Qianjin Zhou, Yu Feng, Sebastian Leptihn, Yunsong Yu, Xiaoting Hua

**Affiliations:** a Department of Infectious Diseases, Sir Run Run Shaw Hospital, Zhejiang University School of Medicine, Hangzhou, Zhejiang, China; b Key Laboratory of Microbial Technology and Bioinformatics of Zhejiang Province, Hangzhou, Zhejiang, China; c Regional Medical Center for National Institute of Respiratory Diseases, Sir Run Run Shaw Hospital, Zhejiang University School of Medicine, Hangzhou, Zhejiang, China; d State Key Laboratory for Managing Biotic and Chemical Threats to the Quality and Safety of Agro-products, Ningbo University, Ningbo, China; e School of Marine Sciences, Ningbo University, Ningbo, China; f Department of Biophysics, Zhejiang University School of Medicine, Hangzhou, China; g Zhejiang University, University of Edinburgh (ZJU-UoE) Institute, Zhejiang University Haining, Zhejiang, China; University of Maryland School of Medicine

## Abstract

Here, we report the complete genome sequence of Vibrio harveyi strain ATCC 33866, generated from Illumina and Oxford Nanopore sequencing. The assembled genome sequence comprises two circular chromosomes with lengths of 3,504,760 bp and 2,218,060 bp, respectively.

## ANNOUNCEMENT

Vibrio harveyi is a notorious zoonotic pathogen infecting marine organisms but also humans (1). Due to the high social and economic burden caused by vibriosis (2), together with the emergence of multidrug-resistant and highly virulent strains (3, 4), it is imperative to gain more knowledge on the biology and genomics of *Vibrio* species in order to develop strategies to combat the pathogen. To date, only six complete genomes of V. harveyi strains are available (ATCC 33843, FDAARGOS_107, WXL538, 345, QT520, and 2011V-1164), and due to this sparsity of genomic data, species identification, comparative genomic analyses, or pathogenesis studies are challenging (5, 6). Here, we report the complete genome sequence of V. harveyi strain ATCC 33866, which will contribute to the genetic data available of the *Vibrio* genus to facilitate further research.

Vibrio harveyi ATCC 33866, isolated from seawater, was purchased from China General Microbiological Culture Collection Center (CGMCCC). It was cultured on a Columbia blood agar plate for 24 h. A single colony was selected and grown in tryptone soya broth at 28°C overnight. Genomic DNA was extracted using the QIAamp DNA Mini kit (Qiagen, Germany) and then analyzed on 1% agarose gel. The same genomic DNA preparation was used for both Illumina and Oxford Nanopore Technologies (ONT) sequencing. Illumina sequencing libraries were prepared by Nextflex rapid DNA sequencing (DNA-Seq) kit. For Nanopore sequencing, libraries were generated using the native barcoding expansion set (EXP-NBD104) and SQK-LSK109 ligation sequencing kit without size selection or shearing. Pooled libraries were qualified and quantified by Qubit 3.0 (Invitrogen, USA) prior to sequencing.

Whole-genome sequencing was performed on the HiSeq X Ten platform (Illumina Inc., USA) as well as on the Nanopore MinION platform (Oxford Nanopore Technologies, UK). Reads were base called using Guppy v5.1.2 (Oxford Nanopore Technologies) with parameters “–flowcell FLO-MIN106 –kit SQK-LSK109 –barcode_kits ‘EXP-NBD104 EXP-NBD114’ and “high-accuracy” was the default mode. Trimmomatic v0.30 was used to trim the Illumina reads (7). Hybrid assembly using the filtered MinION and Illumina reads were assembled with Raven v1.1.10 (8) with error correction (Pilon v1.24 [9]). Default parameters were used for all software unless otherwise specified. The resulting complete genome sequence was annotated by the National Center for Biotechnology Information (NCBI) Prokaryotic Genome Annotation Pipeline (PGAP) (http://www.ncbi.nlm.nih.gov/genome/annotation_prok/).

For Illumina, a total of 4,447,844 150-bp paired-end reads were obtained. For Nanopore, a total of 74,917 reads were obtained, with an average length of 9,527 bp and an *N*_50_ value of 17,008 bp. The depth of Illumina sequencing was on average 126.6 times, while for Nanopore it was 290 times. The assembled genome sequence contains two circular chromosomes (3,504,760 bp and 2,218,060 bp) with a slight difference in GC content (45.0% and 44.9%). The final coverage of the genome is 122.0 x. Annotation showed the genome sequence contains 5,097 protein-coding genes, 34 rRNA (5S-16S-23S rRNA) genes, 130 tRNA genes, 4 noncoding RNA (ncRNA) genes, and 55 pseudogenes (Table 1). Genome data of ATCC 33866 provided here can help to increase our understanding of V. harveyi and *Vibrio* species in general.

**TABLE 1:** Genome features of V. harveyi ATCC 33866

Genome feature	Data for^*a*^:	
	Chr I	Chr II
Size (bp)	3,504,760	2,218,060
GC content (%)	45.0	44.9
No. of rRNAs	31	3
No. of tRNAs	114	16
No. of ncRNAs	4	0
No. of coding sequences	3,175	1,922
Accession no.	CP090179.1	CP090178.1

a Chr, chromosome.

### Data availability.

The whole genome sequence of V. harveyi ATCC 33866 is available in GenBank under the accession numbers CP090179.1 and CP090178.1. The BioSample and BioProject accession numbers are SAMN24371565 and PRJNA791971, respectively. The raw sequence data have been deposited in the Sequence Read Archive (SRA) under accession number SRR18249428 (Nanopore) and SRR17729643 (Illumina).
